# Historical spread routes of wild walnuts in Central Asia shaped by man-made and nature

**DOI:** 10.3389/fpls.2024.1394409

**Published:** 2024-06-06

**Authors:** Xuerong Li, Xiyong Wang, Daoyuan Zhang, Junhua Huang, Wei Shi, Jiancheng Wang

**Affiliations:** ^1^ State Key Laboratory of Desert and Oasis Ecology, Key Laboratory of Ecological Safety and Sustainable Development in Arid Lands, Xinjiang Institute of Ecology and Geography, Chinese Academy of Sciences, Urumqi, China; ^2^ Xinjiang Key Lab of Conservation and Utilization of Plant Gene Resources, Xinjiang Institute of Ecology and Geography, Chinese Academy of Sciences, Urumqi, China; ^3^ College of Forestry and Landscape Architecture, Xinjiang Agricultural University, Urumqi, China; ^4^ Turpan Eremophytes Botanic Garden, Chinese Academy of Sciences, Turpan, China

**Keywords:** *Juglans regia*, SNP, population genomics, gene flow, genetic differentiation

## Abstract

Walnuts have substantial economic value and are of significant interest being a wild-cultivated species. The study has re-sequenced the entire genome of the wild walnut, aligning it with the walnut reference genome, to identify 2,021,717 single nucleotide polymorphisms (SNPs). These were used to examine the genetics of 130 wild walnut samples collected from three countries. Utilizing structural and principal component analysis, the walnut samples from Central Asia were classified into four populations: Ili ah in Xinjiang (I), Dushanbe region in Tajikistan (II), Sary-Chelek, Arslanbob in Kara-Alma regions of Kyrgyzstan (III), and Kok-Tundy region of Kyrgyzstan (IV). The 4 groups showed large differences in nucleotide diversity, population differentiation, and linkage disequilibrium decay, as well as gene flow among them. The present geographic distribution of these populations does not align with the genetic distribution pattern as the populations of Central Asian wild walnuts have experienced similar population dynamics in the past, i.e., the highest effective population size at ca. 6 Ma, two sharp population declines at 6 and 0.2 Ma, and convergence at ca. 0.2 Ma. The genetic distribution patterns are better explained by human activity, notably through archaeological findings of walnut use and the influence of the Silk Road, rather than by current geographic distributions.

## 1 Introduction

The walnut (*Juglans regia* L.) belongs to genus *Juglans* (Juglandaceae) and it is also known as the Persian walnut, and it is a wind-pollinated, monoecious, long-lived, perennial tree that has been domesticated and cultivated worldwide (Manning, 1978, McGranahan and Leslie, 1991, Gunn et al., 2010). The walnut tree is highly valued for its nutritional and medicinal values found in the nuts, as well as for the quality of its wood (Costa et al., 2014; Chen et al., 2019). The natural habitats of walnut wood are confined to the mountainous regions of the Asian continent, stretching from Xinjiang Province in western China to the Caucasus (Akça and Sen, 1994, Hemery et al., 2005; Aradhya et al., 2009). The relationship between the ancient centers of diversity for walnuts and their domestication and subsequent spread by humans remains a subject of considerable debate (Beer et al., 2008; Pollegioni et al., 2017; Bernard et al., 2018). Recent studies of plant domestication history have mostly utilized agronomy, genetics, and archaeobotany (Abbo et al., 2014; Fuller et al., 2014). Quantitative trait locus localization, genome-wide association studies, and genome-wide resequencing studies have identified genes associated with the initial domestication and further diversification of crops (Meyer and Purugganan, 2013; Chomicki et al., 2020). Additionally, the integration of phylogeny, archaeology, and genomics provides a broader insight into the domestication process for various plant species (Wang et al., 2018; Chomicki et al., 2020; Ren et al., 2021). The combination of whole genome resequencing data with fossils and archaeological evidence offers a comprehensive method to review the domestication patterns in cultivated plants (Gaut et al., 2018; Wang et al., 2018), as the case here studied.

*Juglans regia* is thought to be a Neoproterozoic relict of Tertiary forests in Eurasia, where fossil pollen evidence suggests that walnuts have been present since the Pleistocene (Berry, 1912) and that his record persists from the mid-Paleoproterozoic to the Neoproterozoic (Pollegioni et al., 2014). However, the evolutionary history of this species during the Late Tertiary climatic deterioration and Quaternary glaciation is replete with mass extinctions, range reductions, fragmentation, and bottlenecks (Pollegioni et al., 2014; Roor et al., 2017). In addition, the isolation of walnut groups from each other was accelerated by the gradual desertification of Central Asia during the Holocene (Pollegioni et al., 2014). Recent studies have proposed that *J. regia* originated as an ancient hybrid between American and Asian walnut lineages in the late Pliocene (Zhang et al., 2019). Although, man-made walnut is native to the mountains of southeastern Europe and western Central Asia (Bayazit et al., 2007), recent research suggests that Xinjiang Province in China could also be the origin of *J. regia* (Pollegioni et al., 2014). The relationship between the ancient centers of diversity for walnuts and their domestication and subsequent spread by humans remains a subject of considerable debate (Zhang et al., 2000). Human activities have had a profound impact on the distribution and range expansion of walnuts throughout history (Popov, 1929). There is a substantial historical evidence of humans’ close association with walnuts. Dried walnut seeds have been found in the Near East (6230–5790 yr. BP) (Wilkinson et al., 2012), Central Asia (5149 yr. BP) (Mani, 2008), and along the Yellow River Basin in northeastern China (7300 yr. BP) (Rong-Ting, 1989). The consumption of walnuts, valued for their edibility and religious significance, dates back to Persia (7,000 BCE) (Vahdati, 2013), and was facilitated by trade networks such as the Silk Road (Janick, 2002). Despite the long history of use, the domestication of walnuts is relatively recent, with the improvement of asexual varieties of Persian walnuts since the middle of the 20th century (Beer et al., 2008; Feng et al., 2018). Plant cultivation and domestication is a spatially and temporarily dynamic multi-stage process, and taking populations of wild plants to cultivated forms requires human intervention to ensure survival (Gupta, 2004; Fuller, 2007; Fuller et al., 2010). Generally, some reproductive methods or fruit characteristics of perennial plants are changed in this process, but this is not evident in walnuts, due to the fact that the reproductive practices of walnuts under artificial conditions do not differ from those of wild walnuts (Carrion and Sanchez-Gomez, 1992; Gupta, 2004; Fuller, 2007; Fuller et al., 2010; Miller and Gross, 2011a).

The advancement of molecular markers, including Randomly Amplified Polymorphic DNA (RAPD), simple sequence repeats (SSR), amplified fragment length polymorphism (AFLP), expressed sequence marker SSR (EST-SSR), and genomic SSR markers (gSSR), has significantly enhanced the study of plant genetic diversity and structure (Fjellstrom and Parfitt, 1994; Nicese et al., 1998; Potter et al., 2002; Bayazit et al., 2007; Yuan et al., 2018). The study of population genetic structure is essential to analyze the adaptive evolution and genetic relationships of walnut populations across various regions (Gunn et al., 2010). Single nucleotide polymorphisms (SNPs), defined as any single base substitution/indel in the genome of an individual (Primmer et al., 2002), represent next-generation sequencing technologies. SNPs have gained popularity due to the reduced cost of next-generation sequencing, their abundance in the genome, and their ability to express a rich amount of genetic information (Dong et al., 2010; Pecoraro et al., 2016). The walnut genome sequenced, SNP markers can be utilized for rapid and highly automated genotyping, making them ideal for genetic diversity studies and genomic selection analyses.

Central Asia, a region encompassing both the distribution area of wild walnuts and a historically crucial segment of the ancient Silk Road, is of great significance for the study of wild walnut genetic diversity which is important for the conservation and utilization of the walnut species. In this study, we sequenced 130 wild walnuts samples from Central Asia, including Xinjiang China, the Wild Fruit Forest Reserve of Kyrgyzstan, and the Dushanbe region of Tajikistan, using whole genome sequencing technology and walnut reference genomes to elucidate the genetic relationships and genetic diversity in these regions. Further, our aim was to elucidate the genetic relationships and genetic diversity within these regions and to describe the spatial distribution of walnut genetics in Central Asia, considering both geo-historical variability and the impact of human activities throughout history.

## 2 Materials and methods

### 2.1 Plant materials and DNA extraction

**Figure 1:**
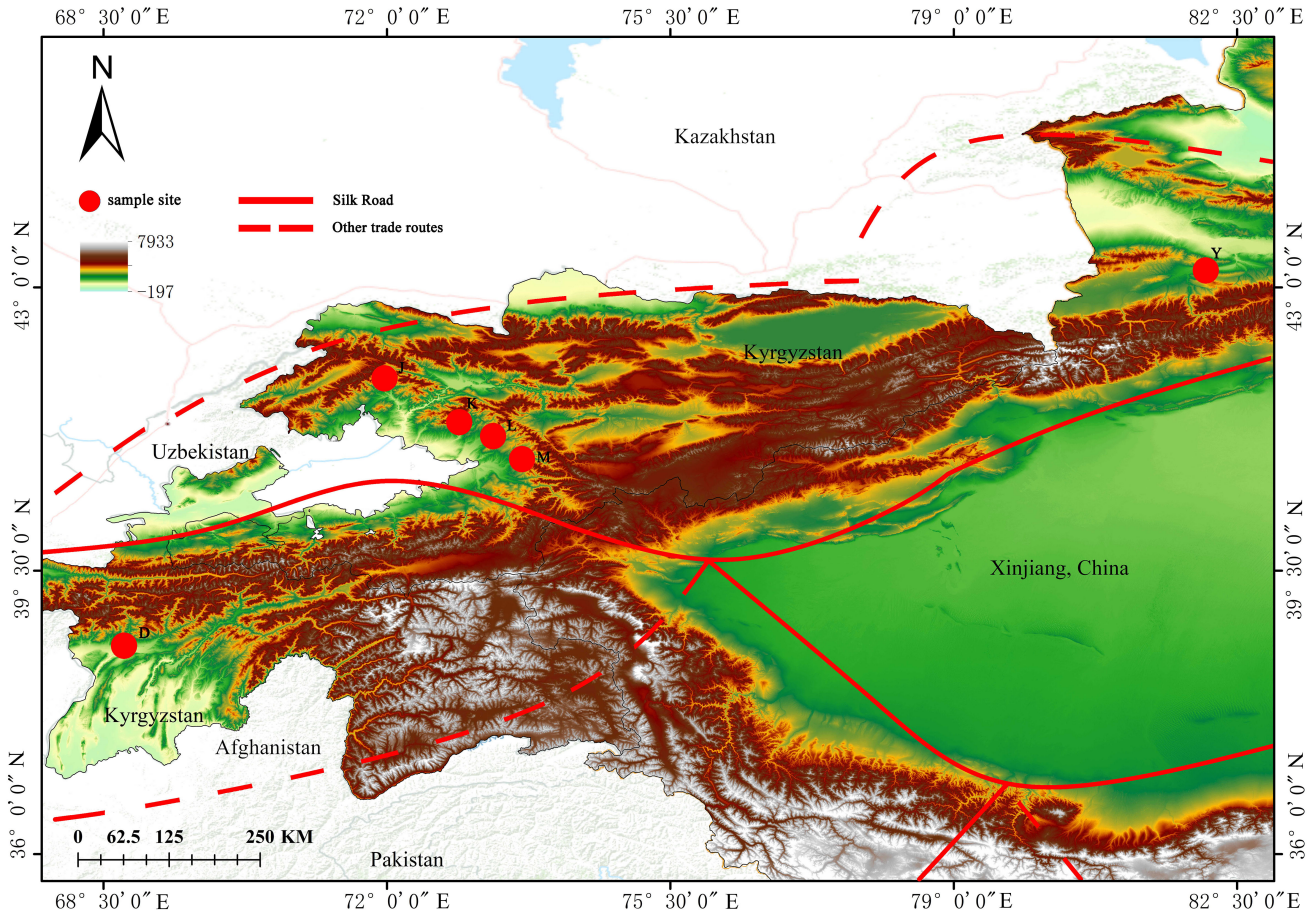
Map of walnut collection areas. The drawing of the Silk Road in the figure is based on the route proposed by Francis et al. (Francis et al., 2006) and the route mapped by UNESCO (UNESCO, 2024).

Samples were collected from large natural walnut forests in the mountains or along forest roads. A minimum of 20 meters was maintained between sampled trees, and a total of 130 fresh young leaf samples from healthy *J. regia* (**Table 1**) were collected. Of the 130 samples, 27 came from Yili, Xinjiang, China (Group Y). 83 samples were gathered from four key wild fruit forest reserves in Kyrgyzstan: 21 samples were collected from the first area, Sary-Cheek (Group J); 21 samples were collected from the second area, Arslanbob (Group K); 26 samples were collected from the Kok-Tundy Area C (Group L), and the last 15 samples came from the Kara-Alma area (Group M). Additionally, 20 samples were obtained from Dushanbe, Uzbekistan (Group D). The fresh leaves were dried in silica gel and stored for future analysis. The geographical distribution of all sampling points is plotted by ArcGIS v10.2 (ESRI Inc., Redlands, California, USA) (**Figure 1**).

**Table 1:** The 130 walnut samples from three regions of Central Asia.

Sampling Site	Country	ID	N	Latitude	Longitude	Elevation (m)
Yili, Xinjiang	China	Y	27	43.217701	82.152833	1533
Dushanbe	Tajikistan	D	20	38.837793	68.959988	1752
Sary-Chelek	Kyrgyzstan	J	21	41.85481	71.964071	1778
Arslanbob	Kyrgyzstan	K	21	41.339877	72.889697	1652
Kok-Tundy	Kyrgyzstan	L	26	40.853421	73.66128	1319
Kara-Alma	Kyrgyzstan	M	15	41.173196	73.311407	1264

DNA extraction was performed using the improved CTAB method (Zhao and Woeste, 2011). Since high-quality gDNA is required in the RAD genotyping technique, we evaluated the concentration and purity of the extracted DNA. These assessments were determined by the ratios of absorbance at 260/280 nm and at 260/230 nm, using a NanoDrop ND-2000 spectrophotometer (Thermo Fisher Scientific, Waltham, MA, USA). This crucial procedure ensured high-quality samples and comparable DNA concentrations for subsequent analysis.

### 2.2 Sequencing and sequencing quality control

For library preparation, DNA was isolated from fresh leaves with 2% cetyltrimethylammonium bromide (CTAB) according to a previously published protocol. A TruSeq library was prepared using the KAPA Hyper Prep Kit (Illumina^®^ platforms, KAPA BIOSYSTEMS, Boston, MA, USA. Cat No. KK8504) following the manufacturers’ manual. All the libraries were sequenced by the HiSeq X^®^ platform (Illumina^®^, San Diego, CA, USA) according to the manufacturer’s instructions. The genome samples were cleaved using the restriction endonuclease EcoRI, and then each one was physically fragmented to construct DNA libraries, and we selected a library of 200–400 bp fragments for insertion. The raw number is obtained by reading 150 bp from the opposite end. The HiSeq X-Ten platform (Illumina, Inc., San Diego, CA, United States) was used for adaptor ligation and DNA cluster preparation.

The original sequencing data contained joint information, low-quality bases, and undetected bases, which would greatly interfere with subsequent information analysis. To obtain clean reads and prevent further interference, the following filters were used: (1) remove reads containing splice sequences. After the first round of quality control using FastQC (version 0.11.5), DNA sequencing reads were trimmed and filtered using Trimmomatic (version 0.36.5). (2) Remove pairs of paired reads whose N content in a single-end sequencing read was more than 10% of the length (3) remove pair of paired reads when a single-end sequencing read contained more than 50% low-quality (<= 5) bases. Perform the above operations based on FastQC to ensure the quality of the trimmed data.

### 2.3 SNP calling and filtering

We used Burrows-Wheeler alignment (BWA) V0.7.17 software (Li and Durbin, 2009) (parameters: mem -t 4 -k 32 -M) to map clean reads to the walnut reference genome (Jregia.genome_v1.0) (Zhang et al., 2020). Use the SAMtools48 (version: 0.1.18) software to convert mapping results to BAM format and filter unmapped and non-unique readings. SAMtools and Picard are used for sorting and removing duplicates, respectively. We removed duplicates of the comparison results with the SAMTOOL (Li et al., 2009) software (Parameters: rmdup), and duplicate reads were filtered using the Picard package (picard.sourceforge.net, version: 1.87).To ensure high quality data, we utilized SAMTOOL (Li et al., 2009) to perform strict filtering of SNP calls based on the following criteria: (1) a minimum SNP support number (coverage depth) of more than 3, (2) a minimum allele frequency of more than 0.05, (3) a deletion rate of less than 10%. Variant identification, programed to detect SNPs, was conducted with the Unified Genotyper application of the Genome Analysis Toolkit (DePristo et al., 2011). GATK was used to construct the general variant call file and merge the gVCF files to form a single variant call file. In order to obtain high-quality SNPs, we first filtered the merged VCF data using the GATK hard filter. Using the ANNOVAR package for genome-based annotation, SNPs were categorized into exonic regions (overlapping coding exons), splice sites (within 2 bp of splice junctions), 5′UTRs and 3′UTRs, intronic regions (overlapping introns), upstream and downstream regions (upstream or downstream of the transcriptional start site by 1 kb), and intergenic regions. SNPs in coding exons were further categorized into synonymous SNPs (which do not cause amino acid changes) and non-synonymous SNPs (which cause amino acid changes; mutations that cause stop-gain and stop-loss are also included in this group), and indels in exonic regions were classified according to the presence or absence of frameshift (3 bp insertion or deletion) mutations.

### 2.4 Structure analysis and phylogenetic tree linkage disequilibrium analysis

To eliminate the potential effect of physical linkage between variants, we selected high-quality SNPs with no more than 20% missing data, and based on this, we refined the loci to ensure that no two loci were located within the same 2000 bp region. To investigate genetic structure among the walnut populations, we conducted a principal component analysis (PCA) using PLINK v1.09 (Purcell et al., 2007) The principal components were also plotted against each other using R3.4 to visualize patterns of genetic variation, and the first three eigenvectors were plotted in 3D space. Additionally, we utilized a hierarchical Bayesian modeling using the STRUCTURE software; this involved a burn-in of 10,000 iterations and running 100,000 iterations with 10 replicates. Subsequently, we clustered the individuals according to K = 1–8 using STRUCTURE v. 2.3.4 (Pritchard et al., 2000); this process utilized an admixture model with associated allele frequencies. The optimal value of K was determined using STRUCTURE HARVESTER v.0.6.94 (Earl and VonHoldt, 2012) according to the Delta K method of Evanno (Evanno et al., 2005), Ln (D|K), and the final posterior probability of K (Pritchard et al., 2000). IQTREE (version 1.6.9) (Lam-Tung et al., 2015) was used to perform phylogenetic analyses to construct maximum likelihood (ML)-based phylogenetic trees based on the GTR + F + R5 model, and 1000 fast bootstrap replicates were performed to determine branching confidence values. After evaluating 130 DNA models, ModelFinder was used to estimate the best-fit model, and finally an ML phylogenetic tree was constructed based on intergenic region SNPs. We used PHYLIP software v 3.696 to produce a cladogram based on the genetic distance matrix using the p-distance formula (Retief, 2000). The phylogenetic tree mapping was done by Figtree using the Neighborhood Joining (NJ) method.

### 2.5 LD analysis and genetic diversity analysis

We calculated the pair-wise LD as r^2^ for each population and within chromosomes using Plink v1.09 following the formula proposed by Hill and Robertson (Hill and Robertson, 1968). The LD attenuation calculation was based on the r^2^ between two SNPs and the distance between two SNPs, and the LD decay was similarly determined based on the r^2^ between two SNPs and the distance between the two SNPs. The r^2^ statistics was obtained using the PLINK software (version 1.07) (Purcell et al., 2007). For each SNP pair, bins of 100 kb were created based on pairwise physical distance. The nucleotide diversity (θπ) of the six groups and genetic distance (FST) were calculated in stepping windows 40 kb in size by VCFtools v0.1.13 (Li, 2011). The horizontal axis represents the position of the genome, and the vertical axis represents the θπ value. The population θπ value reflects the base diversity of the population genome.

### 2.6 Estimated effective population size

To reconstruct the historical demography of the Asian walnuts, we used PSMC (Li and Durbin, 2011). We filtered sequencing data by the following conditions: 1) a mean genome coverage of 18, 2) a per-site filter of 10 reads, and 3) no more than 25% of missing data were followed (Nadachowska-Brzyska et al., 2016). The parameters in PSMC were set to a mass adjustment of 50, a minimum localization mass adjustment of 20, a minimum depth adjustment of one-third of the average depth genome coverage, and a maximum depth adjustment of 2 times. In line with existing literature, the time from birth to offspring was set at 20 years (Bai et al., 2018). The annual mutation rate (u) was estimated to be 5.6×10–^10^ nucleotide substitutions per year using Ka. This converts to 1.12×10–^8^ per generation (20 years).

## 3 Results

### 3.1 Sequencing and SNP variation

After filtering the sequencing data, we obtained high quality clean data, including sequencing data yield, sequencing error rate, Q20 content, Q30 content, GC content and other data. A total of 130 samples were sequenced in this batch, yielding a high sequence quality and volume of data (611.42 Gb). The lowest raw data output of a single sample was 3.46 G, and the highest raw data output was 6.38 G, while the average Effective Rate(%) was 99.83. The mean value of the proportion of bases with mass values >= 20 (Q20) was 97.18%, and the average of the proportion of bases with mass values >= 30 (Q30) was 92.38%. The average GC Content(%) was 37.19. The lowest Effective Rate of a single sample was 97.80, and the highest was 99.95. The lowest Q20 (%) for individual samples was 95.07, and the highest was 97.64. The lowest Q30 (%) for individual samples was 88.34, highest was 93.38. The lowest GC Content (%) for individual samples was 36.39, and the highest was 38.98. GC Distribution is normal (**Supplementary Table 1**). The average depth for the 130 samples was 7.99, with an average Coverage of 1X of 94.53% and an average Coverage of 4X of 78.81%. Over 90% of sequenced reads from the walnuts aligned with the reference genome (**Supplementary Table 2**). The sample comparison rate reflects the similarity between the sample sequencing data and the reference genome, and the coverage depth and coverage can directly reflect the homogeneity of the sequencing data and the homology with the reference sequence. The results show that their similarity to the reference genome meets the requirements for resequencing analysis.

**Figure 2:**
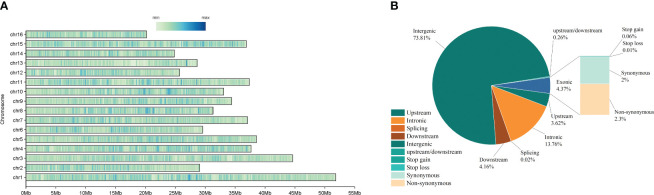
**(A)** Proportion of various SNP in wild walnut and reference genomes.; **(B)** Location of SNP loci on chromosomes.

**Figure 3:**
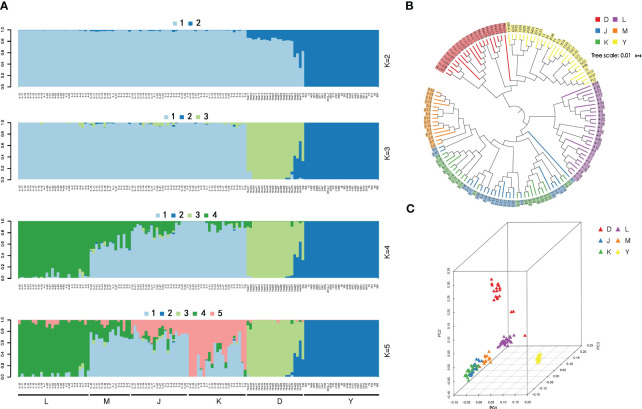
**(A)** Bayesian inference of population structure for 130 wild walnut samples using STRUCTURE. Each box plot within the different colored segments represents a different population. **(B)** Principal Coordinate Analysis (PCA) 3D plot of the 130 *Juglans regia*. The red color indicates walnut populations from the Dushanbe region, the yellow color is from walnut populations in the Walnut Gorge region of Ili, and the blue, green, purple, and orange color is from the four protected wild fruit forests of Kyrgyzstan. **(C)** Neighbor-joining phylogenetic tree based on a genetic distance matrix representing the grouping of 130.

Our SNP detection analysis revealed a total of 2021717 SNP variant loci. Of these, 73090 (3.62%) SNPs in Upstream, 84110 SNPs in Downstream (4.16%), and 1492191 SNPs in Intergenic (73.81%). Additionally, 5304 (0.26%) SNPs were found in 1 Kb region upstream of the gene and 1 Kb downstream of the other gene (Upstream/Downstream). There were 88,425 (4.37%) variants located in the exonic region, of which 1,226 (0.06%) were in Stop gain, 198 (0.01%) in Stop loss, 40,447 (2%) in Synonymous, and 46,554 (2.3%) in Non-synonymous. There were 278,186 SNPs located in Intronic, 367 (0.02%) in Splicing, and 149,191 (73.81%) in Intergenic (**Figure 2A**; **Supplementary Table 3**). The distribution of SNP loci on the chromosome is shown in **Figure 3A**, where the denser distribution of loci is displayed with darker shades, and less dense regions are shown with lighter shades (**Figure 2B**). ANNOVAR functionally annotates genetic variants detected in multiple genomes, enabling subsequent correlation analyses based on the availability of data on the chromosome where the variant is located, the start site, the stop site, the reference nucleotide, and the variant nucleotide.

### 3.2 Phylogenetic relationships and population structure of walnut

The STRUCTURE analysis showed that at K = 2 the whole panel could be divided into two groups: the Ili walnut ditch area formed one, and the remaining areas clustered together as the other group (groups D, K, J, M, L). When K = 3, the Dushanbe walnut group emerged as one group (group D), while the walnuts of the Kyrgyzstan area were still the other (groups L, M, J, K, groups). When K=4, the wild walnuts of the Kyrgyzstan region (groups L, M, J, K) were separated from the wild walnuts of the Kok-Tundy region. When K=5, the distinction between the three remaining Kirghizstan regions is not obvious. (**Figure 3A**).

The PCA corroborated our findings regarding the population structure of wild walnuts, and based on the results of STRUCTURE, it showed a clear structure separation of the panel. We utilized the PCA to analyze the relationship between groups in different places as a complementary method to visualize their clusters. This revealed four well-separated clusters among 130 samples of wild walnuts. The largest cluster contains 51 *J. regia* accessions, and the second cluster mainly includes samples of Ili wild walnuts. The third contains samples of wild walnuts from the Kok-Tundy region of Kyrgyzstan, and the fourth major contains samples of wild walnuts from the Dushanbe region. The findings of both STRUCTURE and PCA revealed that the wild walnut population in Xinjiang was strikingly distinct and unique from other populations. Furthermore, that data indicated obvious gene flow between the walnuts in the three regions of M, J, and K (**Figure 3C**.).

In our study, we assessed the genetic relationships and calculated pairwise genetic distances among over two million Single Nucleotide Polymorphisms (SNPs) employing neighbor-joining tree analysis. Utilizing the genetic information from all 130 samples, we constructed a phylogenetic tree that was categorized into two principal groups. The first group encompassed wild walnuts from the Ili and Dushanbe regions, while the second group was comprised of wild walnuts originating from four distinct wild fruit forest reserves in Kyrgyzstan (**Figure 3B**.). The phylogenetic trees we developed provide a vivid illustration of the genetic affinities among these six geographically diverse regions. A striking observation was that the wild walnuts from Xinjiang demonstrated a closer genetic affinity to those from the Dushanbe region, despite their significant geographic separation. This result is congruent with the classification outcomes derived from both STRUCTURE and PCA analyses, suggesting a robust genetic relationship that transcends geographical barriers.

### 3.3 Genetic diversity and linkage disequilibrium in the genomes of walnuts

**Figure 4:**
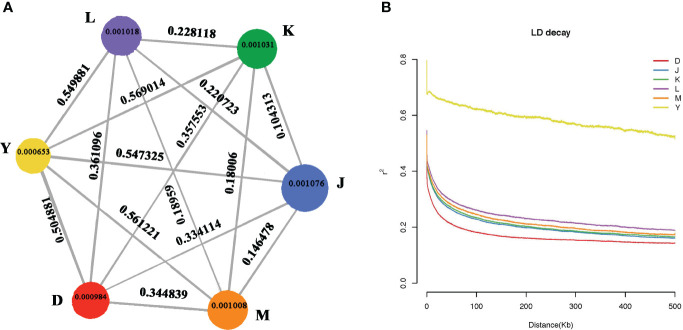
**(A)** Summary of nucleotide diversity (θπ) and population divergence (FST) across groups. The size of each circle represents the nucleotide diversity (θπ) for the group, and values on the line between pairs indicate the population divergence (FST). **(B)** Decay of linkage disequilibrium (LD) in different walnut groups. The abscissa represents the distance of linkage disequilibrium (LD), and the ordinate is the correlation coefficient of linkage disequilibrium.

The observed diversity patterns revealed genetic disparities between walnuts from different regions. Nucleotide diversity (*θπ*) of walnut reveals genetic diversity in different regions. Xinjiang had the lowest nucleotide diversity (*θπ* = 0.0065) and Sary-Cheek had the highest nucleotide diversity, (*θπ* = 0.001076). Further, the results showed that the lowest genetic diversity was found in the wild walnut population from Xinjiang, and the highest genetic diversity was found in the Sary-Cheek population (**Figure 4A**.). The fixation index (FST) is an effective measurement of differences across populations by reflecting the allelic heterozygosity level within a population. The FST index of Y & K is the highest at 0.569014 and also the FST index of Xinjiang, China (Group Y) and other groups show high values all above 0.5. However, the FST index for K & J is the lowest at 0.10313, and all the FST indexes between the Kok-Tundy Area C (Group L), Arslanbob (Group K), Sary-Cheek (Group J), Kara-Alma area (Group M) groups show lower values. Genome level comparisons show that the wild walnut population from Xinjiang has the highest genetic differentiation as compared to the other regions while the lowest genetic differentiation was between the Arslanbob (Group K) and Sary-Cheek (Group J) groups (**Figure 4A**.).

In plant-forward genetics studies, determining the linkage disequilibrium (LD) pattern is essential. Factors such as the population size of the species, fertility (selfing or hybridization), selection pressure, and the rate of recombination all affect the LD level. We estimated the LD decay for each population as a function of physical distance. Then, we sorted SNP pairs in 100 kb-bins based on the distance between pairs and estimated the average r 2 values for each bin. The walnut tree populations from different localities exhibited differing linkage disequilibrium distances (LD) and linkage disequilibrium attenuation (**Figure 4B**.). Wild walnuts from the Dushanbe region reached 0.2 r^2^ at about 100 kb, wild walnuts from the Sary-Cheek (Group J), Arslanbob (Group K), and Kara-Alma area (Group M) regions reached 0.2 r^2^ at 300–400 kb, and wild walnuts from the Kara-Alma area (Group M) region reached 0.2 r^2^ at 500 kb. Wild walnuts from the Ili region were more heterogeneous, with their r^2^ values failing to reach half of the value up to 500 kb. Moreover, the r^2^ values were higher, showing a stronger linkage imbalance. Notably, the wild walnut population from the Dushanbe region of Tajikistan showed a much shorter average distance for LD to decay to 50% of the maximum compared to endemic walnuts. In contrast, the slowest LD decay was in the wild walnut population from Ili, China.

### 3.4 Demographic history

**Figure 5:**
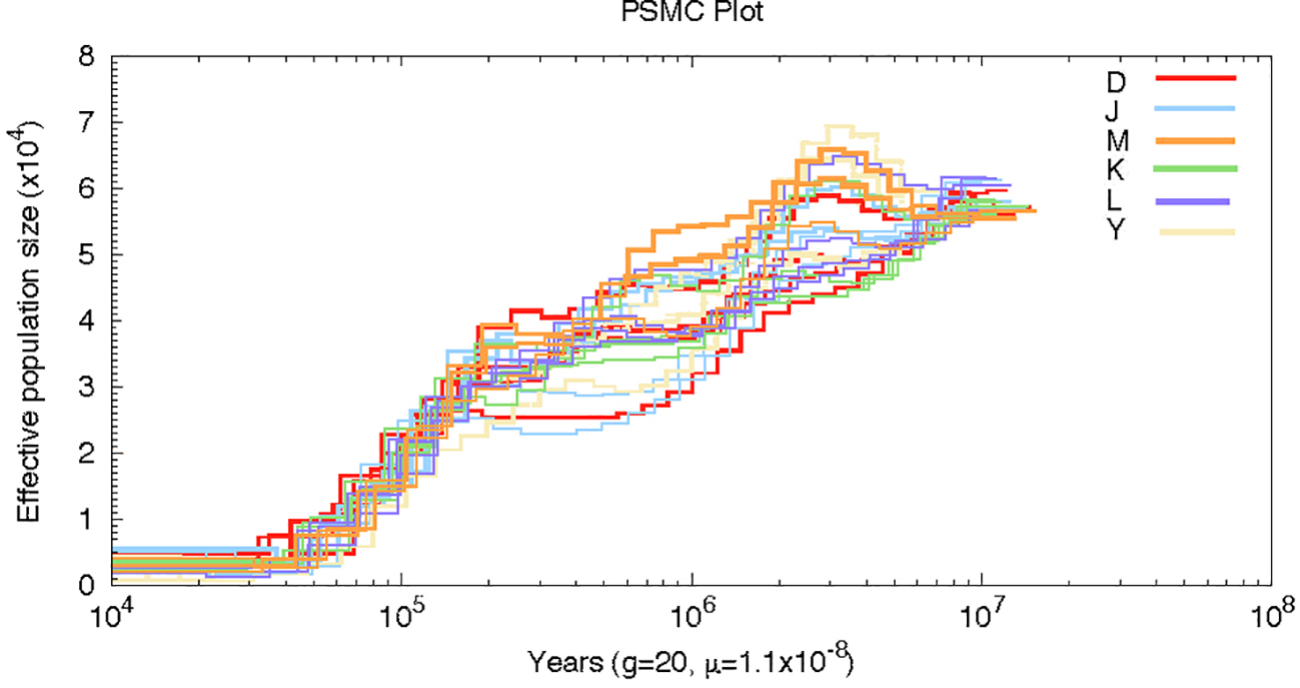
Pairwise sequentially, Markovian coalescent (PSMC) estimates of the changes in the effective population size over time for 20 individuals of Persian walnut.

By employing the pairwise sequential Markovian coalescence, PSMC, we inferred the demographic histories of four representative individuals from each of the six groups in our sampling sites. Twenty-four individuals from these six sampling sites yielded similar inferred population histories, although the number of effective groups prior to 0.2 Ma was not the same. Our analysis revealed that the effective population size (Ne) was higher around 6 Ma and underwent two large declines at a later time. The first decline occurred at about 2 Ma, followed by a period of stability, and then a more dramatic decline at 0.2 Ma. Interestingly, the Persian walnut Ne curves converged at 0.2Ma; this convergence is likely reflecting the time when all sources of *J. regia* last shared a common ancestor (**Figure 5**).

## 4 Discussion

The publication of a high-quality walnut genome advances research on walnut and other related valuable species (Zhang et al., 2020). This includes structural analysis, genetic map construction, and molecular marker-assisted breeding (Liang et al., 2023; Yang et al., 2023; Zhou et al., 2023). However, until an updated reference genome is published, there is limited information available about the genome (Jin et al., 2022; Luo et al., 2022). Sequencing the walnut genome has enabled us to identify SNPs efficiently. This study identified 2021717 SNPs, and these variants provide useful genomic resources for future genetic differentiation studies. Genetic diversity is crucial for the long-term survival of species. The higher the genetic diversity (Wen et al., 2010), the greater the adaptation of the species to the environment, and conversely, the greater the risk of extinction as adaptation declines (Wang et al., 2023).This study found relatively low genetic diversity in walnuts from the Ili and Dushanbe regions and generally high genetic diversity in walnuts from the four Kyrgyz regions of ary-Cheek, Arslanbob, Kok-Tundy, and Kara-Alma. This difference in genetic diversity levels suggests distinct origins of walnuts from the Ili or Dushanbe regions and that of the four regions of Kirghizstan. This hypothesis is consistent with the findings of other recent studies (Torokeldiev et al., 2019; Gaisberger et al., 2020).

Genetic diversity is influenced by a multitude of factors, such as reproductive mechanisms, genetic mutations, natural selection, and the isolation of populations, which can be exacerbated by environmental shifts and anthropogenic interference (Wen et al., 2010; Lu et al., 2020; Perrino and Wagensommer, 2022). Wind pollination, coupled with self-incompatibility, can inadvertently enhance the likelihood of inbreeding, potentially diminishing the heterozygosity of alleles within a population (Wen et al., 2010). This, in turn, may result in a lack of sufficient heterozygosity to maintain high genetic diversity (Wen et al., 2010). Our sampling map indicates that the four regions of Kyrgyzstan were sampled in close proximity to each other. This reduced geographic isolation could be a contributing factor to their higher genetic diversity. The results also show large differences in prior differentiation among populations. The highest index of genetic differentiation was found between the wild walnut populations in Ili, China while lower indices of genetic differentiation were observed among the four regions of ary-Cheek, Arslanbob, Kok-Tundy, and Kara-Alma. Genetic differentiation between species is influenced by a variety of factors, such as gene flow, reproductive practices, and different natural environments. Fragmentation and isolation can lead to restricted gene flow and increased inbreeding in many tree species (Manners et al., 2013).

As a relict plant of the Tertiary period, extensive fossil research suggests that the ancestor of the walnut was widely distributed across Asia and Europe from the Miocene era. This evidence extends from the Late Tertiary to the Quaternary period, with distribution sites extending from Europe to South Asia in the east (Berry, 1912; Renault Miskovsky et al., 1984; Roiron, 1991; Kvacek et al., 1994; Erdei and Magyari, 2011; Yao et al., 2011; Zhang and Sun, 2011; Ivanov et al., 2012; Shatilova et al., 2014). Based on other studies, it is presumed the Persian walnut has survived in LGMs in western Europe, Xinjiang Province, and northeastern to southwestern China over a broad geographic area from 30°N to 45°N (Aradhya et al., 2017; Pollegioni et al., 2017; Feng et al., 2018). During the Pleistocene period, population differentiation may have been influenced by repeated extinctions and colonization during the Quaternary Climate Oscillation (Feng et al., 2018). Thus, the Late Miocene and Pleistocene climatic changes were distributed in broadly similar but fragmented environments across Asia. This fragmented distribution has likely contributed to the walnut’s regionally adaptability, with its effective population size continuously limited by climatic fluctuations and its own long generation time (Zhang et al., 2000; Pollegioni et al., 2017; Roor et al., 2017; Feng et al., 2018). This result is also reflected in the effective population size dynamics in the PSMC plot. The Himalayan movement, marked by the collision and convergence of the Indian plate with the Eurasian continent, significantly influenced the formation of the Tien Shan mountain basin structure (Liu, 2005; Jinyi et al., 2006; Guochao et al., 2008). The formation of the Tibetan Plateau and the Tien Shan’s geographic structure both affect the local climate pattern. As a result, the geographical segregation of the walnut populations along the Tien Shan has become increasingly pronounced (Zhang et al., 2000; Xu et al., 2010). Based on the results of our Structure, PCA, and phylogenetic tree, these 130 wild walnut materials from Central Asia were classified into four major taxa. The wild walnuts from the Ili region were the first to be separated in Structure, whereas the four regions of Kyrgyzstan, Sary-Cheek, Arslanbob, Kok-Tundy, Kara-Alma were not as distinctly separated. The Structure results also indicated that walnuts from four regions, Sary-Cheek, Arslanbob, Kok-Tundy, and Kara-Alma, showed heterozygous genetic information, suggesting gradual genetic infiltration between walnuts from the four regions.

PCA and Phylogenetic Tree analysis indicate that the walnut populations from the Ili region and Dushanbe region are more closely related while the genetic composition of walnut in the Kirghizia regions are more complex. Thus, the genetic spatial structure can’t be explained by using purely geographic space. Given the extensive history of human utilization of walnuts, and the clear historical record of human dispersal of walnuts (Christian, 2000; Vahdati, 2013), it is essential that our structural analysis of the genetic structure of walnuts incorporates the influence of anthropogenic factors. Walnut shells were found in a grave site in the South Caucasus (4,230–3,790 B.C., Late Neolithic) (Wilkinson et al., 2012) and in the vicinity of wheat and barley grains in the Kashmir region (2,700–2,000 B.C.) (Pokharia et al., 2018). Further, nutshells were found in the remains of the 3200-year-old Barikot in the Swat Valley of Pakistan deal (Spengler et al., 2021). Research suggests that walnuts were first domesticated in the Iranian-Anatolian region and then spread westward and eastward by humans (De Candolle, 1855; Pollegioni et al., 2015, 2017; Ding et al., 2022).

Archaeological evidence from the Xinjiang region suggests the utilization of walnuts in Xinjiang can be dated back to the Early Iron Age (seventh to third centuries B.C.) A small number of peach kernels, apricot kernels, and other relics were found in the disturbed soil layer of M1 of the Alagou cemetery in Urumqi (510 B.C.-390.B.C.) (Xiaohong et al., 2014). Emperor Wu of the Han Dynasty came to Central Asia to fight the Xiongnu by connecting the ancient routes in 138 B.C. and 119 B.C. This act opened the first step of the northern route of the Silk Road (Christian, 2000). The rise of the Silk Road (French, 1998; Janick, 2003) facilitated the widespread trade in walnuts across Asia and Europe (Aubaile, 2012; Vahdati, 2013). A number of sites containing walnuts have been discovered in the silk road following the emergence of the Han Dynasty and in the Tarim Basin side of the North-South-East edge (Wang, 1983; Zhang, 1983; Cheng, 1994; Archaeology, 1995; Archaeology and Museum, 1997; Wu et al., 2002; Li et al., 2013; Wang et al., 2020). This evidence indicates that walnuts were widely planted in the Xinjiang region during the Han Dynasty.

According to the above information, the use of walnuts in the Xinjiang region was limited before the Han Dynasty as few walnut artifacts were unearthed from that time. However, a large number of walnut artifacts were unearthed since the Han Dynasty, and walnuts should have been domesticated by human beings before the Silk Road (Mishra, 2020). Thus, with the emergence of the Silk Road, walnuts were widely cultivated and utilized in Xinjiang. Although walnuts are crops, they do not meet the criteria of being domesticated as they can grow normally even when left out of human influence. Over the course of thousands of years of history, humans selectively bred walnuts by choosing suitable seedlings from different geographical locations (Carrion and Sanchez-Gomez, 1992; Harris, 2001; Gupta, 2004; Fuller, 2007; Molnar et al., 2011; Miller and Gross, 2011b). Therefore, we must consider human impact on walnut genetic space as well as the influence of pre-existing geographic differentiation.

Reviewing paleoecological data in southern Europe, Syria, Kyrgyzstan, China, and the Himalayas, suggests that Juglans may have survived the last ice age (Bottema, 1980; Kovar Eder et al., 2006; Beer et al., 2008; Filipova Marinova et al., 2010; Mercuri et al., 2013; Pollegioni et al., 2014; Aradhya et al., 2017). According to results from the PSMC, during the Quaternary glacial period (1–2 Ma), the effective population sizes of Juglans declined due to glacial-induced conditions and have maintained a decline since then. Notably, they converged around 0.2 Ma, potentially sharing a common ancestor. However, the formation of genetic spatial patterns observed in this study may be more influenced by human activities than natural factors. Results from Structure, PCA, and phylogenetic tree analyses for walnuts from four regions of Kirghizia show that although the four sites were collected in close proximity to each other, walnuts from Kok-Tundy could be isolated independently. In contrast, those from the other three sites could not. Similar findings have been reported in studies using SSR (Torokeldiev et al., 2019).

The wild walnut populations in Kirghizstan existed for at most 2,000 years (Beer et al., 2008). Walnuts from different regions met in Kirghizstan, and the Silk Road further influenced the genetic space of wild walnuts there. Anthropogenic impacts, likely stemming from the extensive cultivation of common walnuts for nut production and for reforestation and afforestation initiatives, have been documented since the 5th century AD (Khanazarov et al., 2003). Although the results of the developmental trees indicate that walnuts from the Ili and Dushanbe regions are closely related, there is no clear evidence that the walnuts from Ili and Dushanbe have existed here since the Tertiary period. This uncertainty is partly attributable to the limited availability of fossil walnut records in Tajikistan (Dodonov and Baiguzina, 1995; Dodonov et al., 2006).

One thing is certain: despite human activities, the walnut species is still evolving (Miller and Gross, 2011a). Our results suggest that geographic isolation plays a significant role in the differences between walnut populations. In particular, the wild walnut population in the Ili region has low genetic diversity and faces under high selection pressure, compared to high divergence results in other regions. In contrast, the wild walnut population in the Dushanbe region, which is closely related to Ili, was not subjected to strong selection pressure. Given the characteristics of walnut reproduction, including wind-borne and seed propagation, and their long-lived generations (McGranahan and Leslie, 1991), this selection pressure may have existed for an extended period. Therefore, we need to consider a deeper perspective to conserve the existing wild walnut forests. We could use existing natural conditions to select specific adaptive traits, but the strong anthropogenic influence on walnuts in Central Asia necessitates a different approach. It may be more informative to compare these wild walnuts with modern cultivars to select traits that have been domesticated by humans (GWAS analysis) to elucidate the reasons for the current distribution pattern of wild walnuts in Central Asia.

## 5 Conclusions

This study analyzed the genetic structure of wild walnuts in Central Asia by utilizing SNP markers. Our results revealed high levels of genetic diversity in Central Asia. Specifically, we found that wild walnuts from Ili and Dushanbe are more closely related to each other and likely share a similar origin. These populations differ in origin from walnuts from the four regions of Kyrgyzstan, yet there is evidence of gene flow with each other. Our findings also support the previous view that wild walnut was geographically isolated by Quaternary autochthonous and climatic changes. Furthermore, the widespread use of the wild walnut in Central Asia by humans, particularly after the emergence of the Silk Road, led to greater anthropogenic disturbances and contributes to the present genetic pattern.

## Data availability statement

The data presented in the study are deposited in the National Center for Biotechnology Information (NCBI) repository, accession number PRJNA1072832 and PRJNA1107910.

## Author contributions

XL: Data curation, Methodology, Resources, Visualization, Writing – original draft, Writing – review & editing. XW: Formal Analysis, Investigation, Writing – original draft. DZ: Project administration, Supervision, Writing – original draft. JH: Software, Writing – review & editing. WS: Writing – original draft, Writing – review & editing, Conceptualization, Data curation, Funding acquisition, Methodology, Resources, Supervision. JW: Writing – review & editing, Conceptualization, Formal Analysis, Funding acquisition, Project administration, Software.

## Supplementary material

Supplementary Table 1 130 samples sequenced data output.

Supplementary Table 2 130 Sequencing Sample Comparison and Coverage Statistics.

Supplementary Table 3 Statistics of SNP detection results.
